# Hepatitis E Virus Genotype 7 RNA and Antibody Kinetics in Naturally Infected Dromedary Calves, United Arab Emirates

**DOI:** 10.3201/eid2609.191758

**Published:** 2020-09

**Authors:** Victor M. Corman, Peter Nagy, Stefanie Ostermann, Jacqueline Arloth, Anne Liljander, Rajib Barua, Aungshuman Das Gupta, Fatima Hakimuddin, Judit Juhasz, Ulrich Wernery, Christian Drosten

**Affiliations:** Charité-Universitätsmedizin Berlin, Berlin, Germany. (V.M. Corman, C. Drosten);; German Centre for Infection Research, Berlin (V.M. Corman, C. Drosten);; Emirates Industries for Camel Milk and Products, Dubai, United Arab Emirates (P. Nagy, R. Barua, A. Das Gupta, J. Juhasz);; Institute of Experimental Immunology, Lübeck, Germany (S. Ostermann, J. Arloth, A. Liljander);; Central Veterinary Research Laboratory, Dubai (F. Hakimuddin, U. Wernery)

**Keywords:** hepatitis E virus, viruses, genotype 7, dromedary calves, foodborne infections, food safety, One Health, hepatitis, hepatitis E, viral hepatitis, epidemiology, antibody kinetics, zoonoses, United Arab Emirates

## Abstract

Orthohepevirus A genotype 7 is a novel zoonotic variant of hepatitis E virus. To clarify infection in the animal reservoir, we virologically monitored 11 dromedary dam–calf pairs. All calves became infected during the first 6 months of life and cleared the virus after an average of 2 months. Dams did not become infected.

Infection with hepatitis E viruses (HEVs) is one of the major causes of acute hepatitis in humans (*1*). Most HEV strains infecting humans belong to the virus species *Orthohepevirus A* (HEV-A) (*2*). HEV-A comprises 8 genotypes; genotypes 1–4 and 7 are found in humans. HEV-A genotypes 1 and 2 seem to be restricted to humans. The other 3 genotypes have also been detected in animals, including pigs (genotypes 3 and 4) and camelids (genotype 7) (*1*).

The most likely source of human zoonotic HEV infection is consumption of contaminated food. Typically, human HEV infections lead to acute and self‐limiting disease or asymptomatic seroconversion, but chronic hepatitis E has also been reported, mainly in transplant recipients (*3*,*4*). Infection with camel-associated HEV-A genotype 7 was reported in a patient from the United Arab Emirates with chronic hepatitis after liver transplantation (*4*,*5*). This infection was likely acquired through consumption of contaminated camel products. Despite the risk for zoonotic transmission, data about shedding and immunity of HEV-A genotype 7 infection in naturally infected dromedaries are scarce.

## The Study

We investigated HEV-A RNA and specific antibody levels in dromedary calves and corresponding dams from 1 farm at monthly intervals over the course of the calves’ first year of life. We included serum samples from 11 dam–calf pairs in the United Arab Emirates. The farm contained ≈4,500 camels. The 11 dam–calf pairs investigated in this study were kept in different fenced compartments within 100–150 m of each other but were housed together with other dam–calf pairs in the same paddock throughout lactation (*6*). All calves were born during June 2014. Serum samples were obtained during the first week and then at monthly intervals until 1 year after birth.

**Figure 1:**
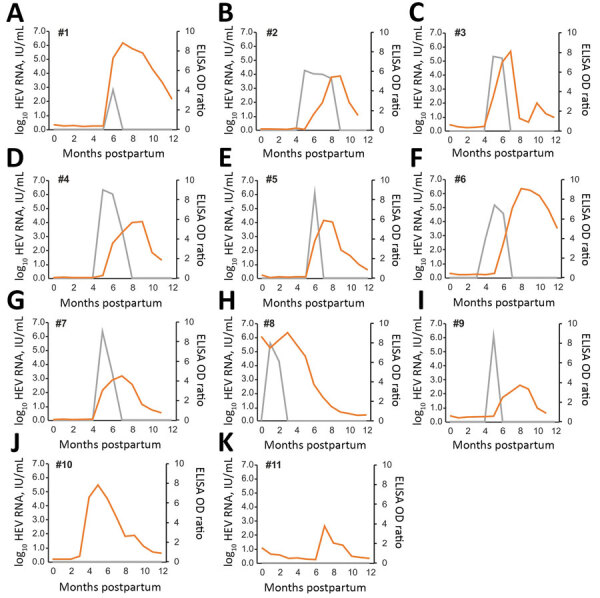
HEV RNA and HEV antibodies in 11 dromedary calves, United Arab Emirates. Gray lines indicate HEV RNA concentration in serum, and orange lines indicate antibody levels. HEV, hepatitis E virus; OD, optical density.

We tested samples for HEV RNA by using 2 reverse transcription PCRs (RT-PCRs) and for HEV antibodies by an HEV-A genotype 7 IgG ELISA (Appendix). In the studied cohort, all calves were naturally infected by HEV-A, as confirmed by RNA detection in serum samples, or seroconversion. In 9 of the 11 calves (#1–#9) (Figure 1), HEV-A RNA was detected in >1 serum sample. In 2 calves (#10 and #11) no RNA was detected, but an increase in ELISA ratio, equivalent to seroconversion, confirmed recent HEV-A infection.

The average age for infection of calves was 4.6 months (range 1–6 months). All HEV-A–RNA positive calves cleared the virus from their blood and showed accompanying seroconversion (Figure 1). Average viremia was 2.1 months (range 1–4 months). Viral RNA concentrations in serum samples ranged from 6.6 × 10^2^ IU/mL to 2.3 × 10^6^ IU/mL (mean 4.6 × 10^5^ IU/mL).

**Figure 2:**
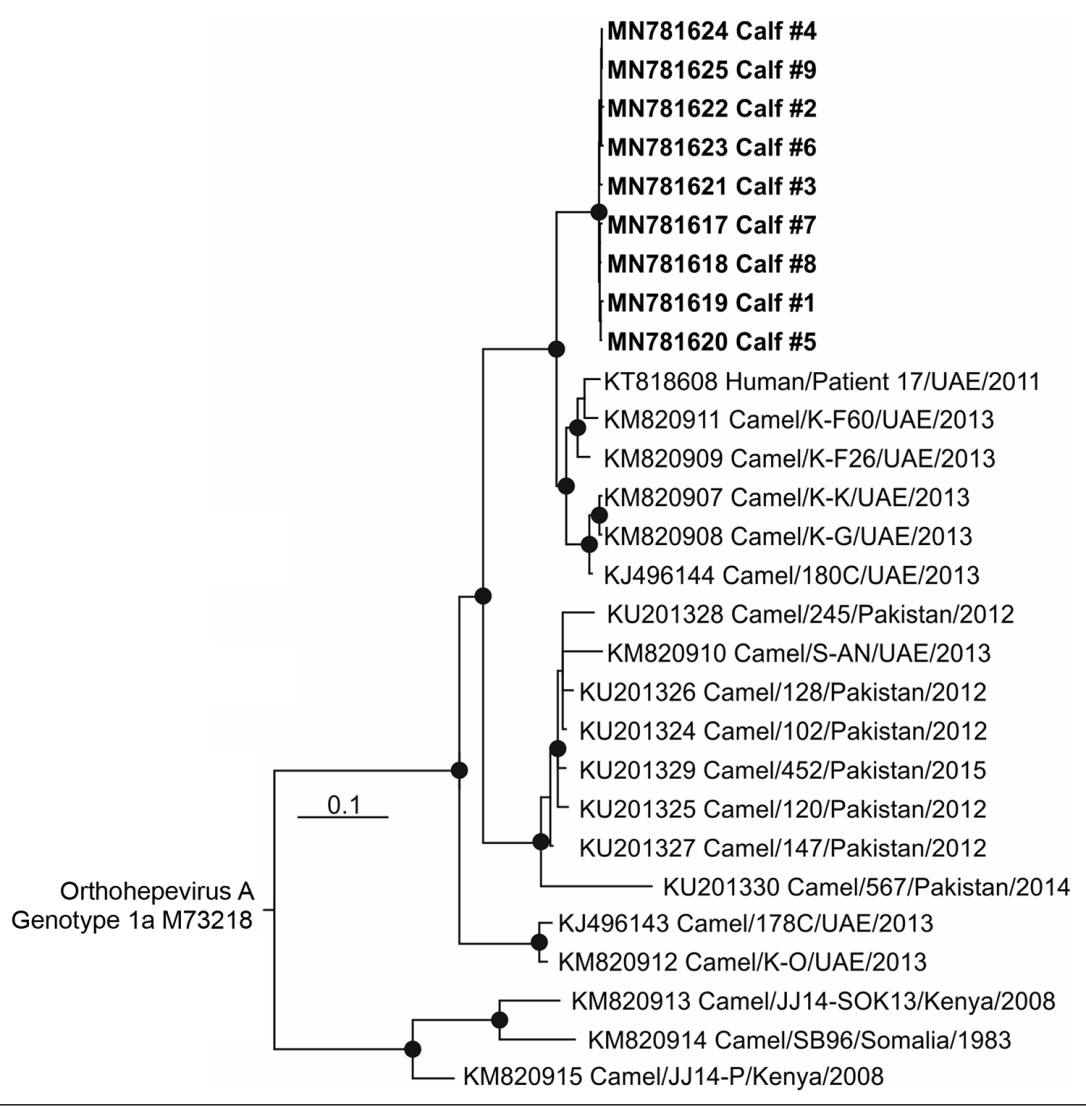
Phylogenetic tree of open reading frame 1 of HEV RNA sequences from 11 camel calves (bold), United Arab Emirates, and other *Orthohepevirus A* genotype 7 sequences available in GenBank as of May 1, 2019. The tree was calculated by using MrBayes (http://mrbayes.sourceforge.net) and a generalized time reversible substitution model. One million generations were sampled every 500 steps, and 20% of trees were discarded as burn-in. Bold indicates sequences obtained during this study. An *Orthohepevirus A* genotype 1 sequence (GenBank accession no M73218) was used as the outgroup. Taxon names of all reference sequences include GenBank accession number, strain, country of origin, and year of sampling. GenBank accession numbers for sequences obtained in this study are MN781617–25. Scale bar indicates nucleotide substitutions per site.

Phylogenetic analysis of partial open reading frame 1 region sequences confirmed that all strains belong to HEV-A genotype 7 (Figure 2). Sequences described clustered with a clade of HEV-A sequences obtained from camels and a human patient, all from the United Arab Emirates. All new sequences were highly similar and had 0–3 nt exchanges in the 283-nt fragment, which is consistent with the epidemiologic link of all infections and might indicate a common source of infection on this farm.

We compared novel sequences with all other camel associated HEV-A genotype 7 sequences available in GenBank (as of June 1, 2019). The novel sequences differ by 6.0% to 20.6% nt content within the partial open reading frame 1 region.

We found that none of the corresponding serum samples from dams were positive for HEV-A-RNA at any time or showed seroconversion during the study. We also found that 6/9 dams showed reactivity at the time of parturition by using the applied HEV-A genotype 7 ELISA and remained reactive through the year, and the other 3 dams remained nonreactive during the study.

ELISA reactivity for all calves decreased after infection, and 2 of the 11 calves became negative (#8 and #11) (Figure 1). These 2 calves were the ones that had earliest infection date and the longest time span between infection and last available serum sample. To what extent the decrease of antibody levels is associated with a decreasing immunity against HEV-A genotype 7 was not investigated but should be the subject of future studies.

The observed natural course of HEV-A genotype 7 infection in camels is similar to that for HEV-A genotype 3 in pigs. Pigs represent the zoonotic source of most human HEV infections in the Northern Hemisphere (*7*). In piglets, HEV-A genotype 3 infection usually occurs at the age of 2–3 months, coinciding with the decrease in levels of maternal antibodies (*8*). HEV-A RNA is present in blood for »1–2 weeks, but longer periods <12 weeks have been described (*9*). Similarly, in our study of camels, all calves became infected within the first 6 months, and the duration of the viremia was an average of 8 weeks. Prevalence of HEV RNA in pigs at slaughter, which usually takes place at the age of 5–8 months, was found to be high, with <10% viral RNA detection in blood and <50% viral RNA detection in feces (*7*,*10*,*11*). As camels are slaughtered at higher age (»2 years of age even in industrial farming) (*12*), a lower risk for HEV transmission associated with meat production might apply to camels in comparison with pigs.

The absence of RNA detection in dams suggests immunity in adult animals. The lack of detected HEV infection in 3 seronegative dams, despite close contact with their infected calves, points to additional factors, such as a T-cell–mediated immunity that might protect against HEV infection. This hypothesis is emphasized by simultaneous detection of antibodies and virus RNA in some calves and suggests that not all antibodies provide sterile immunity. This finding becomes essential when one considers intervention strategies, such as vaccination. Nevertheless, the time of seroconversion coincides with the time of decreasing viremia and infection clearance. A similar pattern of virus versus antibody findings has been found for pigs, humans, and hares (*8*,*13*–*15*).

The observation that all investigated calves were infected in the first year of life indicates a highly active enzootic infection pattern. The virus seems to be widespread in the studied herd and could therefore be widely associated with dromedaries. Because calves have contact with other calves on farms, the infection cycle is probably maintained by calf-to-calf transmission. This factor is relevant because humans are rarely in contact with camel calves but make intensive use of adult animals and derived products. However, our study was conducted in an industrial farming context. Different infection patterns with infection at higher age might be observed in husbandries that involve lower densities of animals and might also vary in other husbandry practices.

## Conclusions

We provide essential information regarding age of infection, virus shedding, and immunity for camel-associated, zoonotic HEV-A genotype 7. Knowledge about the distribution of a zoonotic virus in its animal reservoir is needed to mitigate risks of acquisition along the husbandry and production chain. Intervention by vaccination will need to target calves at a time when they are still coreared with dams. Risks for the general human population seem to be low because humans are rarely in contact with camel calves unless directly involved in camel breeding. Future studies should investigate age-associated infection patterns in less industrialized forms of camel husbandry and the actual risk for transmission to humans when animals are slaughtered.

Appendix Additional information on hepatitis E virus genotype 7 RNA and antibody kinetics in naturally infected dromedary calves, United Arab Emirates.
