# Supplementary material for: Hepatitis E Virus Genotype 7 RNA and Antibody Kinetics in Naturally Infected Dromedary Calves, United Arab Emirates
Source: Emerg Infect Dis. 2020 Sep;26(9):2214–7. doi: 10.3201/eid2609.191758 (PMC7454054; doi:10.3201/eid2609.191758)
Supplement: Appendix — Additional information on hepatitis E virus genotype 7 RNA and antibody kinetics in naturally infected dromedary calves, United Arab Emirates. [file 19-1758-Techapp-s1.pdf]

Article DOI: https://doi.org/10.3201/eid2609.191758

Hepatitis E Virus Genotype 7 RNA and Antibody Kinetics in Naturally Infected Dromedary Calves, United Arab Emirates

Appendix

Methods

Reverse Transcription PCR Testing and RNA Quantification

We screened for hepatitis E virus (HEV RNA) by using an HEV reverse transcription PCR (RT-PCR) that amplified a 283-nt fragment of the RNA-dependent RNA polymerase gene as described (*1*). We amplified the 283-nt fragment by using Sanger sequencing (Seqlab; https://www.microsynth.seqlab.de) and analyzed this fragment by using Geneious 11 software (https://www.geneious.com).

For quantification of HEV RNA concentration, we additionally tested all samples by using a real-time RT-PCR that was calibrated on the basis of the World Health Organization International Standard for HEV RNA (*2*,*3*). Oligonucleotide primer sequences used in the real-time RT-PCR were HEV_A-rtF, 5′-GGT GGT TTC TGG GGT GAC-3′; HEV_A_rtR, 5′-AGG GGT TGG TTG GRT GRA-3′; and HEV_A-rtP, FAM-TGA 5′-TTC TCA GCC CTT CGC - MGB-3′. We observed no discrepancies between both assays, confirming the robustness of both test systems for HEV-A genotype 7 detection.

Antibody Detection

We detected HEV-A–specific IgG by using an ELISA and a truncated capsid-protein (amino acid positions 112–660) of HEV-A genotype 7. Antigen was expressed in *Escherichia coli*, purified, and coated on ELISA plates. Goat anti-llama IgG labeled with horseradish peroxidase, which had been shown to be suitable for dromedary camel samples was used as secondary antibody (*4*,*5*). Optimal conditions for ELISA were determined by testing several experimental conditions and using different antigen concentration, ranging from 2 μg/mL to 10 μg/mL, and by using HEV-characterized serum samples tested for HEV antibodies by using an

adapted human HEV ELISA (EUROIMMUN Medizinische Labordiagnostika AG, https://www.euroimmun.com) and the RecomLine Immunoblot (MIKROGEN, https://www.mikrogen.de/start.html) (*4*).

The HEV-A ELISA was put into a ready-to-use diagnostic reagent kit in collaboration with EUROIMMUN. Optical density (OD) was measured at 450 nm and 605 nm. To standardize OD, the OD ratio was calculated by dividing the OD, measured at 450/605 nm, of each sample, by the OD of a reference serum. Therefore, the calculated OD ratios can be used as a relative measure for the concentration of antibodies. Because no defined negative control serum samples were available, a cutoff value (OD ratio 0.72) was calculated by multiplying 3-fold the mean of the OD ratios of calve serum samples obtained ≥2 months after parturition and 1 month before PCR-confirmed HEV infection (7 calves, 18 samples). Camel serum samples were diluted 1:100 before testing.

References

1. Drexler JF, Seelen A, Corman VM, Fumie Tateno A, Cottontail V, Melim Zerbinati R, et al. Bats worldwide carry hepatitis E virus–related viruses that form a putative novel genus within the family *Hepeviridae*. J Virol. 2012;86:9134–47. PubMed https://doi.org/10.1128/JVI.00800-12

2. Baylis SA, Blümel J, Mizusawa S, Matsubayashi K, Sakata H, Okada Y, et al.; HEV Collaborative Study Group. World Health Organization international standard to harmonize assays for detection of hepatitis E virus RNA. Emerg Infect Dis. 2013;19:729–35. PubMed https://doi.org/10.3201/eid1905.121845

3. Girón-Callejas A, Clark G, Irving WL, McClure CP. In silico and in vitro interrogation of a widely used HEV RT-qPCR assay for detection of the species *Hepevirus A*. J Virol Methods. 2015;214:25–8. PubMed https://doi.org/10.1016/j.jviromet.2014.11.025

4. Rasche A, Saqib M, Liljander AM, Bornstein S, Zohaib A, Renneker S, et al. Hepatitis E virus infection in dromedaries, north and east Africa, United Arab Emirates, and Pakistan, 1983–2015. Emerg Infect Dis. 2016;22:1249–52. PubMed https://doi.org/10.3201/eid2207.160168

5. Meyer B, Juhasz J, Barua R, Das Gupta A, Hakimuddin F, Corman VM, et al. Time course of MERS-CoV infection and immunity in dromedary camels. Emerg Infect Dis. 2016;22:2171–3. PubMed https://doi.org/10.3201/eid2212.160382
